# Classification and Clinical Features of Headache Disorders in Pakistan: A Retrospective Review of Clinical Data

**DOI:** 10.1371/journal.pone.0005827

**Published:** 2009-06-08

**Authors:** Muhammed Murtaza, Mehreen Kisat, Haroon Daniel, Aziz B. Sonawalla

**Affiliations:** 1 Medical College, Aga Khan University Hospital, Karachi, Pakistan; 2 Department of Nursing, Aga Khan University Hospital, Karachi, Pakistan; 3 Department of Medicine, Neurology Section, Aga Khan University Hospital, Karachi, Pakistan; National Institutes of Health, United States of America

## Abstract

**Background:**

Morbidity associated with primary headache disorders is a major public health problem with an overall prevalence of 46%. Tension-type headache and migraine are the two most prevalent causes. However, headache has not been sufficiently studied as a cause of morbidity in the developing world. Literature on prevalence and classification of these disorders in South Asia is scarce. The aim of this study is to describe the classification and clinical features of headache patients who seek medical advice in Pakistan.

**Methods and Results:**

Medical records of 255 consecutive patients who presented to a headache clinic at a tertiary care hospital were reviewed. Demographic details, onset and lifetime duration of illness, pattern of headache, associated features and family history were recorded. International Classification of Headache Disorders version 2 was applied.

66% of all patients were women and 81% of them were between 16 and 49 years of age. Migraine was the most common disorder (206 patients) followed by tension-type headache (58 patients), medication-overuse headache (6 patients) and cluster headache (4 patients). Chronic daily headache was seen in 99 patients. Patients with tension-type headache suffered from more frequent episodes of headache than patients with migraine (p<0.001). Duration of each headache episode was higher in women with menstrually related migraine (p = 0.015). Median age at presentation and at onset was lower in patients with migraine who reported a first-degree family history of the disease (p = 0.003 and p<0.001 respectively).

**Conclusions/Significance:**

Patients who seek medical advice for headache in Pakistan are usually in their most productive ages. Migraine and tension-type headache are the most common clinical presentations of headache. Onset of migraine is earlier in patients with first-degree family history. Menstrually related migraine affects women with headache episodes of longer duration than other patients and it warrants special therapeutic consideration. Follow-up studies to describe epidemiology and burden of headache in Pakistan are needed.

## Introduction

Headache disorders are ranked amongst the ten most disabling conditions in the world by World Health Organization (WHO). The global prevalence of active headache diseases in the adult population is 46%. Tension-type headache and migraine are the two most common headache disorders affecting 42% and 11% adults respectively. About 3% of the world's population is affected by chronic headache that lasts for more than 15 days per month[1].

While headache has been an unaddressed cause of morbidity around the world, it has remained largely unrecognized in the developing world[2]. Most clinical and epidemiological studies have originated in developed countries and there is scarce literature to support treatment guidelines or public health interventions to deal with headache in low and middle income countries where 85% of the world's population lives.

The aim of this study is to characterize patients with headache disorders in Pakistan who seek medical treatment. Classification and clinical features as well as predictors of disability have been described.

## Methods

### Study participants and variables

Retrospective review of medical records of all patients who presented to a specialist headache neurology clinic at Aga Khan University Hospital (AKUH) between June 2006 and December 2007 was conducted. AKUH is an urban tertiary care center in Karachi, Pakistan that serves patients from middle and upper socioeconomic groups mostly on out-of-pocket payments. For each patient, a routine clinic questionnaire was completed by a qualified and experienced specialist in headache neurology (the last author, AS) during the initial consultation. The questionnaire recorded demographic details, onset and lifetime duration of illness, pattern of headache, associated factors and family history (see **Table 1** for a summary and see supporting information for the complete form). The International Classification of Headache Disorders version 2 was applied and as many diagnoses as necessitated by the criteria and as clinically justified were assigned to each patient[3]. Migraine with aura was defined as at least 2 episodes of migraine with typical aura. Patients who reported a frequency of greater than or equal to 15 episodes per month were assigned the additional diagnosis of chronic daily headache (CDH)[4].

**Table 1:** Variables recorded in the clinic questionnaire.

**Sociodemographic Information**	Age at first presentation to our clinic
	Gender
	Weight in kilograms
	Height in centimeters
**History of Headache Disorder**	Age at onset
	Duration of symptoms
	Frequency of headache
	Minimum duration of each episode
	Maximum duration of each episode
**Characteristics of headache episodes**	Warning symptoms
	Presence of Aura
	Site of pain - Unilateral/Bilateral
	Triggers/Aggravating Factors
	Relieving factors
**Associated Symptoms**	Photophobia
	Phonophobia
	Vomiting
	Nausea
	Vertigo
	Lacrimation
	Other Associated Symptoms
**Family History of Headache**	Mother/Father/Brothers/Sisters
	Second-degree relatives
**Diagnosis**	Clinical Assessment
	ICHD^1^ Diagnoses

1 ICHD – International Classification of Headache Disorders version 2.

The study protocol was approved by the Ethical Review Committee at Aga Khan University Hospital. The requirement for informed consent was waived by the committee as we extracted data from medical records and no identifiable information was used in this analysis.

### Data processing and statistics

Statistical Package for Social Sciences (SPSS) version 15.0 was used for data entry, processing and statistical analysis at the end of the study period.

Average duration of each headache episode was calculated as a mean of minimum and maximum duration. Family history was stratified into none, first and second degree history. Body mass index (BMI) was calculated using weight and height for patients at least 18 years of age. The patients were divided into underweight, normal, overweight and obese according to WHO recommendations for using BMI to evaluate risk of cardiovascular disease in South Asians[5].

Distributions of all continuous variables were tested for normality using the Shapiro-Wilk test. Distributions of age at presentation, age at onset, BMI, current frequency of headaches, lifetime duration of illness and average duration of each episode were not normal (p<0.01 for each). Therefore, descriptive statistics were calculated and have been reported as median±interquartile range. Mann-Whitney test and Kruskal-Wallis test were used to test differences in continuous variables. Analysis of Co-Variance (ANCOVA) was used where adjustment for covariates was needed and correlations were evaluated using Kendall's tau-b. Chi-square test and odds ratio (OR) were used to test differences in categorical variables. P-values less than 0.05 were considered significant.

## Results

### Characteristics of Study Population

A total number of 255 patients attended this specialist headache clinic in the study period including 169 women and 86 men.

The average age at first presentation of patients was 32±22 years. No significant difference in age at presentation was found between men and women. About 81% of the patients were between 16 and 49 years of age. Body Mass Index (BMI) was calculated for 201 patients and 60% of them were found to be overweight or obese. There was no significant association of frequency of headache, duration of each headache episode or number of associated symptoms with obesity in either gender.

Distribution of type of primary headache across gender is presented in **Table 2**.

**Table 2:** Distribution of Primary Headaches by ICHD type in this study sample (n = 255)^1^.

Headache type (ICHD code)	Women		Men		Both	
	N	%^2^	N	%^2^	N	%^3^
Migraine (1)	141	68.4	65	31.6	206	80.8
Migraine without Aura (1.1)^4^	112	67.5	54	32.5	166	80.6
Migraine with Aura (1.2)^4^	11	31.3	5	68.8	16	7.8
Migraine Complications (1.5)^4^	28	75.7	9	24.3	37	18.0
Childhood Migraine Syndromes (1.3)^4^	1	50.0	1	50.0	2	1.0
TTH (2)	35	60.3	23	39.7	58	22.7
Cluster (3)	0	0.0	4	100.0	4	1.6
Medication-overuse headache(8.2)	3	50.0	3	50.0	6	2.4
Psychiatric Disorder (12)	2	66.7	1	33.3	3	1.2
Cranial Neuralgia (13)	1	33.3	2	66.7	3	1.2
Total	169	66.3	86	33.7	255	100

1 Patients may have one or more headache diagnoses. Totals represent the actual number of males and females included in the study. ICHD-International Classification of Headache Disorders version 2.

2 Percentage expressed as patients of the gender within total number of patients with the type of headache.

3 Percentage expressed as patients with the type of headache over total study population.

4 Percentage expressed as patients with subtype of migraine over total number of migraineurs.

### Migraine

Migraine was the most commonly diagnosed primary headache found in 81% (n = 206) patients. Out of these, 8% had migraine with aura and 81% had migraine without aura. 18% were diagnosed as having a complication of migraine; most commonly chronic migraine. Two patients with childhood periodic syndromes were also seen. 68% of migraineurs were women with a male to female ratio of almost 1∶2. Clinical characteristics at presentation of patients diagnosed with migraine have been summarized in **Table 3**.

**Table 3:** Clinical characteristics of Migraine (ICHD 1.x) patients (n = 206).

Variable	Category	Women		Men		Both	
		N	%^1^	N	%^1^	N	%^2^
Frequency (per month)	<5	45	68.2	21	31.8	66	37.3
	6–14	27	64.3	15	35.7	42	23.7
	>14	48	69.6	21	30.4	70	39.0
Duration (years)	<0.5	10	55.6	8	44.4	18	9.2
	0.5–2	28	66.7	14	33.3	42	21.5
	2–5	26	65.0	14	35.0	40	20.5
	>5	71	74.7	24	25.3	95	48.7
Average Episode Duration (hours)	<2	18	60.0	12	40.0	30	23.4
	2–10	31	58.5	22	41.5	53	41.4
	10–24	12	66.7	6	33.3	18	14.1
	>24	27	100.0	0	0.0	27	21.1
Age at Onset (years)	<16	21	56.8	16	43.2	37	18.9
	16–29	72	71.3	29	28.7	101	51.5
	30–49	34	69.4	15	30.6	49	25.0
	>49	8	88.9	1	11.1	9	4.6
Site of pain	Unilateral	52	70.3	22	29.7	74	35.9
	Bilateral	16	61.5	10	38.5	26	12.6
	Orbital	30	83.3	6	16.7	36	17.5
	Frontal	27	62.8	16	37.2	43	20.9
	Parieto-temporal	28	54.9	23	45.1	51	24.8
	Occipital	29	67.4	14	32.6	43	20.9
	Hemicranial	7	87.5	1	12.5	8	3.9
	Holocranial	32	72.7	12	27.3	44	21.4
	Neck and Shoulders	28	90.3	3	9.7	31	15.0
Quality of pain	Pounding	9	69.2	4	30.8	13	6.3
	Stretching	13	65.0	7	35.0	20	9.7
	Throbbing	48	73.8	17	26.2	65	31.6
	Pressure Like	36	73.5	13	26.5	49	23.8
	Heaviness	9	60.0	6	40.0	15	7.3
Associated Symptoms	Photophobia	80	74.8	27	25.2	107	51.9
	Phonophobia	88	72.7	33	27.3	121	58.7
	Nausea	88	73.9	31	26.1	119	57.8
	Vomitting	52	78.8	14	21.2	66	32.0
	Vertigo/Dizziness	27	77.1	8	22.9	35	17.0
	Lacrimation	5	83.3	1	16.7	6	2.9
Aggravating Factors	Sunlight	16	66.7	8	33.3	24	11.7
	Heat	16	80.0	4	20.0	20	9.7
	Stress	62	66.7	31	33.3	93	45.1
	Lack of sleep	23	67.6	11	32.4	34	16.5
	Sound	23	82.1	5	17.9	28	13.6
	Menstrual cycle	16	100.0	0	0.0	16	7.8
	Excess Light	14	70.0	6	30.0	20	9.7
	Food related	12	54.5	10	45.5	22	10.7
Relieving Factors	Lying down	55	73.3	20	26.7	75	36.4
	Darkness	32	71.1	13	28.9	45	21.8
	Silence	34	75.6	11	24.4	45	21.8
	Local Pressure	14	63.6	8	36.4	22	10.7
	Analgesic Medications	40	65.6	21	34.4	61	29.6
	Sleep	12	63.2	7	36.8	19	9.2
	Restlessness and Pacing	6	66.7	3	33.3	9	4.4
Family History	No Family History	79	69.9	34	30.1	113	54.9
	First Degree Relatives	53	65.4	28	34.6	81	39.3
	Second Degree Relatives	9	75.0	3	25.0	12	5.8

1 Percentage expressed as patients of the gender within total number of patients in the category.

2 Percentage expressed as patients in the category over total number of patients with migraine (n = 206).

**Figure 1:**
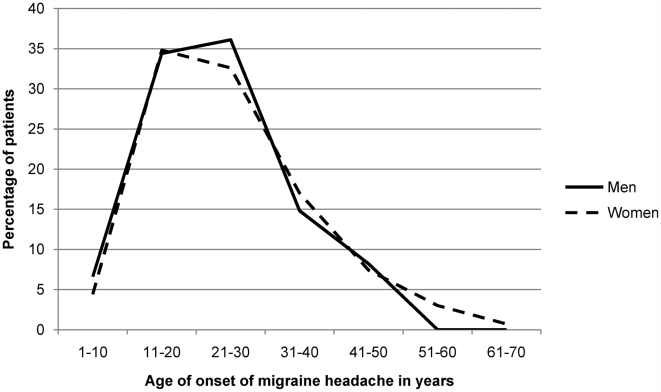
Age at onset of migraine headache (in years) compared across gender: No significant difference is seen.

**Figure 2:**
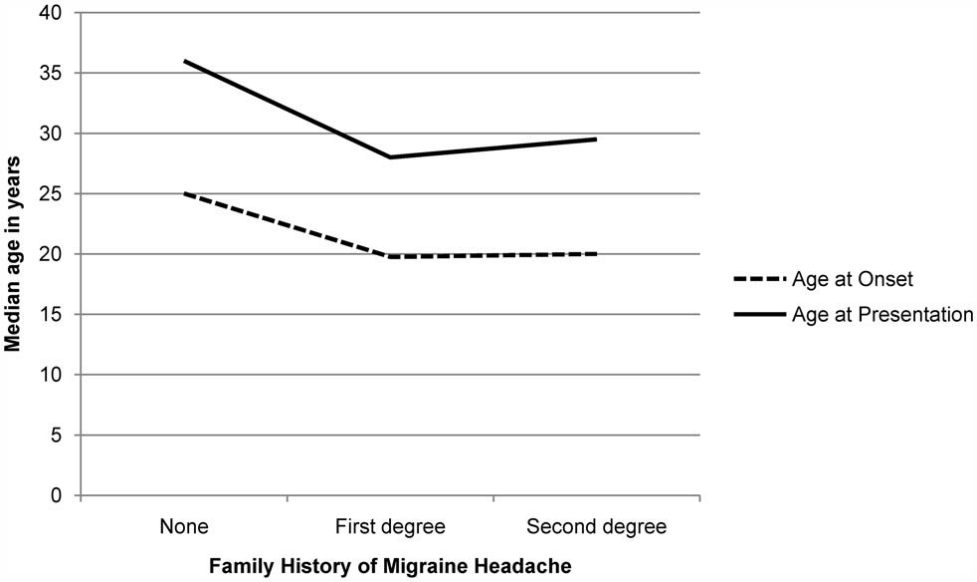
Median reported age at onset and age at first presentation in migraine patients (in years) across family history of disease: Onset and presentation of migraine is significantly earlier in patients with first-degree family history of headache as compared to patients with no such history (p = 0.003 and p<0.001 respectively).

The average age at first presentation of migraineurs was 31.5±19 years while the average reported age at onset of migraine headache was 22±15 years. There were no significant differences in age at presentation and age at onset between men and women (see **figure 1**). However, onset and presentation of migraine in patients with a first-degree family history of headache was significantly earlier than those with no family history (U = 3533.5, p = 0.003, r = −0.19 and U = 2871.5, p<0.001, r = −0.26 respectively; see **figure 2**). Median age at presentation of patients with no family history of migraine was 36±22 years while that of patients with a first degree family history was 28±19 years. Similarly, median reported age at onset of patients without any family history of migraine was 25±17 years while that of patients with a first degree family history was 19.75±10 years. Two patients who were diagnosed with childhood migraine syndromes (ICHD 1.3.x) were not included in this analysis.

Average duration of each episode in hours was significantly higher in women (U = 1179.5, p = 0.003). Women who reported menstruation as a triggering factor were about 4 times more likely to have migraine attacks that last 24 hours or longer than women who did not (95% CI OR: 1.36–12.24, p = 0.012) and about 7 times more likely to have such attacks when compared to all migraineurs (95% CI OR: 2.22–19.68, p = 0.001). No significant differences were observed when triggering factors other than menstruation were considered.

Photophobia, phonophobia, nausea, vomiting, vertigo or dizziness and lacrimation were used to calculate an arbitrary total number of associated symptoms for each patient. Women reported a significantly higher number of associated symptoms than men in this sample (F = 4.79, p = 0.030) even when adjusted for age at presentation, duration of symptoms, BMI and family history of disease. Number of associated symptoms also correlates significantly with lifetime duration of illness and average duration of each migraine episode (Tau-b = 0.111, p = 0.040 and Tau-b = 0.168, p = 0.011 respectively).

Co-occurrence of other types of headache with migraine is presented in **Table 4**.

**Table 4:** Co-morbidity in patients with migraine headaches (ICHD 1.x, n = 206).

Headache type (ICHD code)	Women		Men		Both	
	N	%^1^	N	%^1^	N	%^2^
TTH (2)	16	66.7	8	33.3	24	11.7
Medication-overuse headache(8.2)	3	60.0	2	40.0	5	2.4
Non-vascular Intracranial (12)	1	100.0	0	0.0	1	0.5
Head and Neck Trauma (5)	0	0.0	2	100.0	2	1.0
Total	141	68.4	65	31.6	206	100.0

1 Percentage expressed as patients of the gender within total number of people with the type of headache.

2 Percentage expressed as patients with the type of headache over total number of patients with migraine (n = 206).

### Tension-type headache

Tension Type Headache (TTH) was diagnosed in 23% (n = 58) patients. 12% had infrequent episodic headache, 52% had frequent episodic headache and 28% had chronic headache. 60% (n = 35) patients diagnosed were women. The median age at first presentation was 35.5±23 years and the median reported age at onset of illness was 26±23 years. The median lifetime duration of illness was 4.0±4.5 years and the median current frequency of headaches in these patients was 30±16 episodes per month.

**Figure 3:**
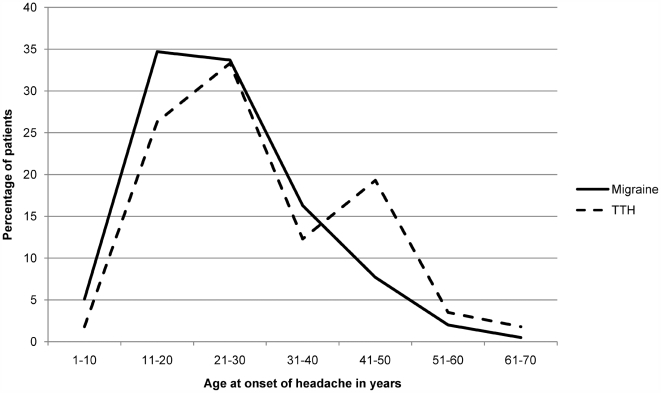
Reported age at onset (in years) of migraine and tension-type headache: Migraine onset peaks in the second and third decade of life and falls later on unlike tension-type headache.

**Figure 4:**
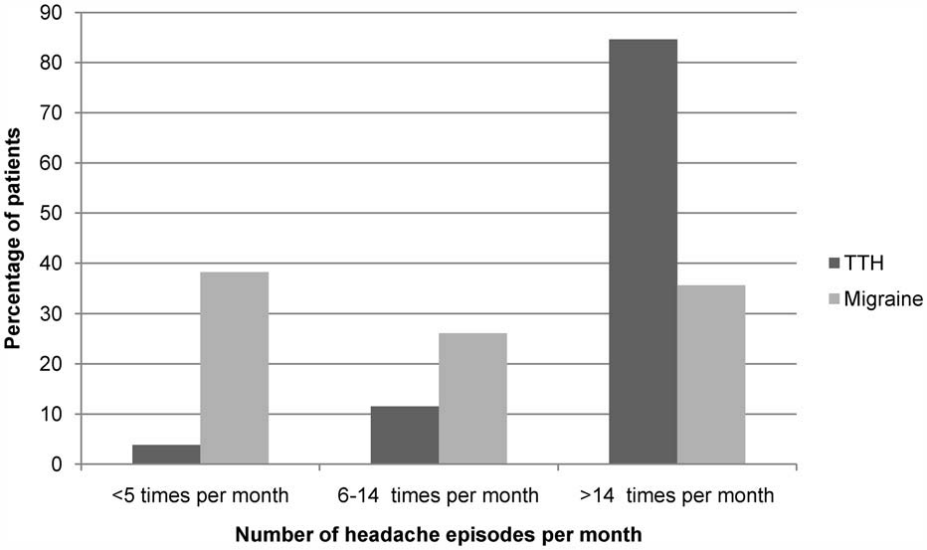
Frequency of headache episodes in migraine and tension-type headache: In contrast with migraine, majority of the patients with tension-type headache suffered from headache on more than 14 days per month. The frequency of headache episodes in patients with tension-type headache was higher than those with migraine (p<0.001).

Patients diagnosed with only migraine had an earlier onset and a longer lifetime duration of illness than patients diagnosed with only tension-type headache (U = 2075.0, p = 0.003, r = −0.19 and U = 2097.5, p = 0.004, r = −0.18; see **figure 3**). On the other hand, the frequency of headache episodes in patients diagnosed with only tension-type headache was higher than those diagnosed with only migraine (U = 956.5, p<0.001, r = −0.33; see **figure 4**).

### Chronic Daily Headache

Chronic daily headache was seen in 39% (n = 99) patients. 64% of these patients were women. 57% patients with chronic daily headache had migraine, 22% had tension-type headache and 13% had both. 5% of these patients were diagnosed with medication-overuse headaches. There was no significant difference in age of presentation, age of onset and gender distribution in the group of patients with chronic daily headache as compared to the rest of our headache clinic population. Obesity was not found to be a significant risk factor for chronic daily headache.

## Discussion

This is the first glimpse of characteristics of headache disorders in Pakistan classified and diagnosed according to ICHD-2. More than 80% of the patients who sought treatment were between 15 and 49 years of age, the most productive age group and majority of these patients were women. Similar gender distributions have been reported previously. Jensen and Stovner reported male to female ratios of 1∶3 and 4∶5 in migraine and tension-type headache respectively and an earlier study from Pakistan reported that all types of headache are over three times more common in women[6], [7].

Migraine and tension-type headache were the two most common presentations in this clinical sample. Epidemiological evidence from around the world suggests TTH is the most common cause of primary headache[1]. However, migraine was seen as the most common reason for presentation to a headache neurologist in our study and published clinical reports agree with this observation. Several possible explanations can be offered for this disparity including under-recognition of primary headache especially TTH as a “real disease” by patients and health practitioners, lower individual morbidity of TTH and the lack of a medical referral system in Pakistan. It can also be speculated that TTH presents less frequently than migraine because of a lower community prevalence. While local epidemiological data is needed to test these explanations, patients with TTH who sought medical care suffered from more frequent episodes of headache that those with migraine in our study. Literature reports suggest that amount of disability associated with TTH on a societal level is much higher than that with migraine especially when measured as absence from work[8]. Increasing awareness and improving the capability of primary care physicians to manage TTH and migraine is likely to help decrease the associated burden.

Menstrually Related Migraine (MRM) was associated with increased morbidity with headache episodes lasting days. In our sample, 11% of female migraine patients suffered from menstrually related migraine. These women are known to have longer lasting headache episodes with increased severity of pain[9]. Hence, menstrually related migraine that presents to primary care needs to be recognized to initiate specific therapy.

First-degree family history of migraine was associated with an earlier onset of the disease. This is consistent with the findings of Rainero et al, who compared the clinical, psychological and pharmacological characteristics of the disease between the two groups of patients and reported that the only significant difference was an earlier onset of disease[10]. It is also in accordance with a study of pediatric migraine patients which showed an earlier onset of migraine in patients with a higher familial impact than in those without a positive family history of the disease[11]. Common migraine is a polygenic disease i.e. several genes have minor contributions to its pathophysiology and genetic predisposition combines with environmental triggers to cause clinical symptoms. The search for genes that predispose to migraine has not yielded uniform results till date most likely due to heterogeneity of patients studied and lack of a reliable endophenotype to classify the disease[12]. Demonstration of the effect of genetic loading on onset of migraine in the Pakistani population is an important finding in this aspect. This is a highly inbred population with a 60% prevalence of consanguinity, over 80% of which are between first cousins[13]. Therefore, genetic studies of migraine conducted in this population may be more likely to demonstrate minor gene effects.

Chronic daily headache was reported by 39% of the patients. Two clinical studies in the South Asian population found a similar or higher prevalence amongst headache patients but the proportions of migraine and tension-type headache differed between the two. Ravi et al reported 37% patients suffered from CDH and tension-type headache was the most prevalent within this group[14]. In an earlier study, Chakravarty reported that almost 50% of headache clinic patients were diagnosed with CDH and 82% of them suffered from chronic migraine followed by 16% from chronic TTH[15]. The latter results are replicated in this study to some extent.

### Limitations

This study characterizes patients with headache disorders who sought medical treatment with a headache neurology specialist. Therefore, it is inappropriate to generalize the results of this study to headache disorders in the community. The concept of a specialist headache clinic is relatively new in Pakistan. Combined with lack of a established medical referral system, most patients who suffer from headaches are unaware of diagnostic and therapeutic options. In addition, patients in this study had financial and physical access to an urban tertiary care hospital and they may not be entirely representative of the general population in the country.

Nevertheless, this study highlights characteristics of headache patients who seek medical treatment and presents factors that predict headache associated morbidity. It is one of the first few clinical reports of headache disorders from South Asia. Local epidemiological evidence is required to guide public health and research policies.
